# The effects of *Bifidobacterium animalis* QC08 on reducing uric acid level and providing renal protection in mice with hyperuricemia

**DOI:** 10.3389/fmicb.2025.1529626

**Published:** 2025-01-30

**Authors:** Huijia Mao, Yang Fan, Fang Tan, Xingyao Long

**Affiliations:** ^1^Collaborative Innovation Center for Child Nutrition and Health Development, Chongqing University of Education, Chongqing, China; ^2^Department of Clinical Nutrition, Chongqing University Jiangjin Hospital, Chongqing, China; ^3^College of Pre-School, Chongqing University of Education, Chongqing, China

**Keywords:** *Bifidobacterium animalis* QC08, hyperuricemia, xanthine oxidase, renal injury, uric acid transporters

## Abstract

**Objective:**

The present study aimed to investigate the uric acid-lowering effects of *Bifidobacterium animalis* QC08 and explore its underlying mechanisms.

**Methods:**

Hyperuricemia (HUA) model in mice was established using potassium oxonate (250 mg/kg) and yeast extract (15 g/kg). The serum levels of uric acid (UA), blood urea nitrogen (BUN), creatinine (Cr), and liver xanthine oxidase (XO) were measured in four groups, including normal group, control group, allopurinol group (5 mg/kg), and *Bifidobacterium animalis* QC08 group (10^10^ CFU/kg) using enzyme colorimetry. Additionally, serum tumor necrosis factor-α (TNF-α) and interleukin-6 (IL-6) levels were assessed using enzyme-linked immunosorbent assay (ELISA). Pathological changes in renal tissue were examined through hematoxylin–eosin (HE) staining.

**Results:**

*In vivo* experimental results indicated that compared with the normal group, the serum UA, Cr, and BUN levels, as well as the levels of inflammatory factors (TNF-α and IL-1β), and the activities of hepatic xanthine oxidase (XOD) and adenosine deaminase (ADA) were significantly elevated in the control group (*p* < 0.05). The expression levels of uric acid transport-related genes (UAT, ABCG2, and OAT1) in kidney tissue were significantly downregulated (*p* < 0.05), and evident kidney damage was found. In contrast, compared with the control group, the *Bifidobacterium animalis* QC08 group exhibited a significant decrease in serum UA, BUN, Cr, TNF-α, and IL-6 levels, along with reduced hepatic XOD and ADA activities (*p* < 0.05). Additionally, *Bifidobacterium animalis* QC08 was found to regulate the mRNA transcription of renal uric acid transporters, leading to significantly upregulation of the expression levels of UAT, ABCG2, and OAT1 genes (*p* < 0.05).

**Conclusion:**

*Bifidobacterium animalis* QC08 demonstrates certain uric acid-lowering, anti-inflammatory, and renal protective effects, which are associated with the inhibition of XOD activity and the modulation of the expression levels of uric acid transporter genes (UAT, ABCG2, and OAT1).

## Introduction

Hyperuricemia (HUA), characterized by an imbalance in the production and excretion of uric acid (UA), has emerged as the second most prevalent metabolic disorder (Xu et al., 2024). HUA is typically defined as a UA level exceeding 420 μmol/L in men and 350 μmol/L in women (Bardin and Richette, 2014). Numerous clinical studies demonstrated that chronic HUA can result in gout, a condition marked by acute or chronic inflammation and joint damage caused by the deposition of monosodium urate crystals (Stamp and Chapman, 2013). It is also an independent risk factor for several conditions, including hypertension, hyperlipidemia, metabolic syndrome, and chronic kidney disease (Chen et al., 2018; Gamala and Jacobs, 2019; Kielstein et al., 2020; Kumar et al., 2022; Wang et al., 2025). HUA poses a substantial risk to human health.

The current pharmacological treatments for HUA primarily encompass xanthine oxidase (XOD) inhibitors, purine nucleoside phosphorylase inhibitors, uricosuric agents, and uricase. Although these medications exhibit significant clinical efficacy, they are also associated with high costs, severe side effects, and low patient tolerance. Including gastrointestinal bleeding, hepatotoxicity, and allergic responses (Cao et al., 2012; Ishikawa et al., 2020; Tausche et al., 2009). Meanwhile, approximately two-thirds of uric acid (UA) is excreted through the kidneys, while the remaining portion is eliminated via the intestines. Therefore, probiotics that are safe, effective, have minimal side effects, and can alter the composition of intestinal flora represent a potential strategy for managing HUA.

Probiotics are live microorganisms that, when taken in appropriate amounts, provide health benefits to the host (Hill et al., 2014), which exert beneficial effects through several mechanisms, involving lowering intestinal pH, reducing the colonization and invasion of pathogenic microorganisms, and modulating the host’s immune responses (Williams, 2010). Studies demonstrated that probiotics can regulate gut microbiota homeostasis, stabilize gastrointestinal barrier function, alleviate gastrointestinal diseases, and exhibit antioxidant, anti-inflammatory, and anti-tumor effects. Additionally, they enhance immune function, thereby playing a positive regulatory role in various diseases. They can aid in the treatment of conditions, such as allergies and obesity (Elisashvili et al., 2019; Vera-Santander et al., 2023; Zucko et al., 2020). Several representative strains of lactic acid bacteria with probiotic potential have been utilized as dietary supplements, including *Lacticaseibacillus rhamnosus*, *Lactobacillus helveticus*, *Limosilactobacillus fermentum*, *Lactobacillus gasseri*, *Lactobacillus delbrueckii subsp. bulgaricus*, *Lactobacillus acidophilus*, *Lacticaseibacillus casei*, and *Limosilactobacillus reuteri*.

Extensive research on probiotics in medical, pharmaceutical and food science fields has shown that probiotics can effectively regulate HUA-induced dysbiosis through intestinal colonization, thereby promoting purine and UA metabolism (Wu et al., 2021). Probiotics can increase the abundance of gut microbiota associated with the production of short-chain fatty acids (SCFAs), thereby enhancing SCFA production. This enhancement inhibits both serum and hepatic XOD activity, thereby inhibiting UA production, ultimately alleviating HUA (Ni et al., 2021). In addition, probiotics exhibit notable antioxidant activity in the host’s gut, promoting the production of antioxidant enzymes and facilitating the elimination of reactive oxygen species. This process consequently alleviates oxidative damage, related to HUA development (Feng and Wang, 2020). Clinical studies have demonstrated that probiotics can effectively reduce UA level in patients with HUA. Specifically, *Ligilactobacillus salivarius* (*L. salivarius*) CECT 30632 exhibited a 100% conversion rate for both inosine and guanosine, along with a 50% conversion rate for UA. Rodríguez et al. (2023) conducted a randomized clinical trial, in which patients with HUA and a history of gout attacks received *L. salivarius* CECT 30632 for six consecutive months. They found a significant reduction in serum UA level, a decrease in the frequency of gout attacks, and improvements in oxidative stress- and metabolic syndrome-associated indicators. Despite its limited scope and inherent constraints, this preliminary clinical trial demonstrated the significant potential of *L. salivarius* CECT 30632 in mitigating HUA and gout. Another study pointed out that patients with HUA who received febuxostat in conjunction with a Bifidobacterium quadruple live bacterial preparation experienced significantly reduced serum UA level. Compared with the control group, which received only febuxostat, the experimental group exhibited significant changes in the abundance of gut microbiota. This finding suggests that the *Bifidobacterium* quadruple live preparation might reduce UA level by modulating gut flora and enhancing bacteria’s ability to degrade UA (Zhan et al., 2020). *Lactobacillus gasseri* (*L. gasseri*) PA-3 has been demonstrated to effectively lower UA level. Kurajoh et al. (2018) conducted a randomized, double-blind, controlled trial, and they found that participants consuming yogurt containing *L. gasseri* PA-3 experienced a reduced rate of serum UA elevation induced by purine nucleotides. Increasing the dosage, extending the duration of use, or combining the treatment with food components that inhibit XOD activity significantly enhances its ability to reduce UA level. Another study demonstrated that yogurt containing *Limosilactobacillus fermentum* GR-3 significantly reduced serum UA level compared with regular yogurt. The mechanism involves regulating the gut microbiota, reducing host inflammatory responses, promoting UA excretion, and protecting both kidney and intestinal functions (Zhao et al., 2022). The potential of probiotics in alleviating HUA has remarkably garnered researchers’ attention. Supplementation of *Bifidobacteria* and *Lactobacilli*, the two wellknown probiotics, was effective to lower UA in mice (Wang H. et al., 2022). *Lactobacillus plantarum* Q7 (Cao et al., 2022) and *L. brevis* DM9218 (Wang et al., 2019) also exhibited promising potentials in reducing UA levels. Moreover, *Bifidobacterium animalis* are also effective, related to the inhibitory effect of animal *Bifidobacterium animalis* on inflammation (Wang J. et al., 2022; Ma et al., 2024).

In this study, the UA-lowering activity of *Bifidobacterium animalis* QC08 was verified in an *in vivo* HUA mice model. Specifically, the study aimed to evaluate the effect of this therapy in underlying HUA alleviation through measurements of changes in the UA synthesis and excretion and pro-inflammatory cytokine levels.

## Materials and methods

### Experimental animals

Male specific pathogen-free (SPF)-grade Kunming mice, weighing 20–25 g, were purchased from the Hunan SJA Laboratory Animal Co., Ltd. (Hunan, China). Mice were housed in a barrier facility for acclimatization under a 12-h light and dark cycle with free access to food and water. This study was approved by the Animal Ethics Committee of Chongqing Functional Food Engineering Technology Research Center (Approval No. 2023042602B).

### Experimental materials

Uric acid test kit (Nanjing Jiancheng Bioengineering Institute, Nanjing, China), potassium oxonate (Aladdin Holdings Group Co., Ltd., Shanghai, China), mouse interleukin-1 beta (IL-1β) enzyme-linked immunosorbent assay (ELISA) kit (Invitrogen, Carlsbad, CA, USA), mouse tumor necrosis factor-alpha (TNF-α) ELISA kit (Invitrogen), creatinine (Cr) and blood urea nitrogen (BUN) test kits (Nanjing Jiancheng Bioengineering Institute), and hematoxylin–eosin (HE) staining kit (Shanghai Biyuntian Technology Co., Ltd., Shanghai, China) were utilized.

*Bifidobacterium animalis* QCO8 was preserved at the Guangdong Microbial Culture Collection Center (GDMCC, Beijing, China) under the accession number GDMCC No. 64207. The strain maintained at −80°C was resuscitated. The bacteria was then diluted with 0.9% normal saline to 1.0 × 10^9^ CFU/mL.

### Establishment of HUA mouse model and drug administration

The experimental procedure follows the method described by Bi et al. (2024) with some modifications. After 1 week of acclimatization, 24 male Kunming mice were randomly divided into four groups: normal group, model group, allopurinol group, and *Bifidobacterium animalis* QC08 group (*n* = 6 mice in each group). All drugs were formulated as suspensions in 0.5% sodium carboxymethyl cellulose (CMC-Na). Starting from day 1, all groups except the normal group were administered potassium oxonate (250 mg·kg^−1^) and yeast extract (15 g·kg^−1^) by gavage for modeling, once daily for 14 consecutive days (Fu et al., 2024; Li et al., 2023). On day 15, treatment with corresponding drugs began; the allopurinol group received 5 mg·kg^−1^ allopurinol, and the *Bifidobacterium animalis* QC08 group received 10^10^ CFU/kg of QC08 (Bi et al., 2024), while the normal group was administered 0.5% CMC-Na by gavage for 27 consecutive days. On day 42, mice were fasted overnight, and blood was collected from the orbital sinus 1 h after the last administration. The kidneys and livers of mice were collected, with a portion of the kidneys fixed in 4% formaldehyde for pathological examination, while the remaining liver and kidney tissues were preserved at −80°C for future use.

### Enzyme colorimetric method for detecting serum biochemistry in mice

Blood was collected from the orbital sinus of mice, maintained at room temperature for 30 min, and then centrifuged at 4,500 r·min^−1^ for 10 min to obtain the serum. The levels of UA, Cr, and BUN were measured using Varioskan LUX (Thermo Fisher Scientific, Waltham, MA, USA) following the instructions of the kits.

### Enzyme colorimetric method for measuring XOD and ADA activities in mouse liver

A portion (0.1 g) of the mouse liver was homogenized in 1 mL of tissue lysis solution using an ice bath, followed by centrifugation at 8,000 r·min^−1^ for 10 min at 4°C. The supernatant was collected and kept on ice for the measurement of XOD and adenosine deaminase (ADA) activities using Varioskan LUX (Thermo Fisher Scientific, Waltham, MA, USA) according to the instructions of the kits.

### Measuring serum TNF-α and IL-6 levels in mice utilizing ELISA

The serum levels of TNF-α and IL-6 were measured using Varioskan LUX (Thermo Fisher Scientific, Waltham, MA, USA) according to the instructions of the ELISA kits.

### HE staining for observing pathological changes in mouse kidneys

Kidney tissues were fixed in 4% formaldehyde solution, followed by routine paraffin embedding and sectioning to a thickness of approximately 6 μm. The HE staining was performed, and the morphological changes in the kidney tissues in each group of mice were observed under a light microscope (Olympus BX43F, Japan).

### Quantitative reverse transcription polymerase chain reaction (qRT-PCR) for measuring relative mRNA expression in kidney tissues

RNA from liver and muscle tissues was extracted using the Trizol reagent (Gan et al., 2021). Complementary DNA (cDNA) was subsequently synthesized using HiScript SuperMix (Vazyme, Nanjing, China). qRT-PCR was performed using the StepOnePlus real-time PCR system (Thermo Fisher Scientific, Waltham, MA, USA) and a fluorescent dye (SYBR Green PCR Master Mix). Table 1 presents the primer pairs utilized in the study. The target gene expression levels were analyzed using the 2^−ΔΔCT^ method. Beta-actin was selected as an internal reference gene (Li et al., 2019).

**Table 1:** Primer sequences.

ABCG2	Forward primer	5′-CTGGCCTTAATGCTATTCTGG-3′
	Reverse primer	5′-TTGAAATGGGCAGGTTGAGGT-3′
UAT	Forward primer	5′-TGAGGAAGGAGGGTATGTGGT-3′
	Reverse primer	5′-GAAGCAAAGCTCAAAGGGCAT-3′
OAT1	Forward primer	5′-CTGATGGCTTCCCACAACAC-3′
	Reverse primer	5′-GTCCTTGCTTGTCCAGGGG-3′
GAPDH	Forward primer	5′-ACCCAGAAGACTGTGGATGG-3′
	Reverse primer	5′-GAAGCAAAGCTCAAAGGGCAT-3′

### Statistical analysis

Statistical analysis was conducted using SPSS 26.0 software (IBM, Armonk, NY, USA). Data were presented as mean ± standard deviation (x̄ ± s). Group means were compared using one-way analysis of variance (ANOVA). For comparing groups with homogeneous variances, the least significant difference (LSD) method was employed, while Dunnett’s T3 test was used for comparing groups with heterogeneous variances. The t-test was utilized for making comparisons between two independent samples. A *p*-value of less than 0.05 was considered statistically significant.

## Results

### Effects of *Bifidobacterium animalis* QC08 on mouse body weight changes and kidney index

**Figure 1:**
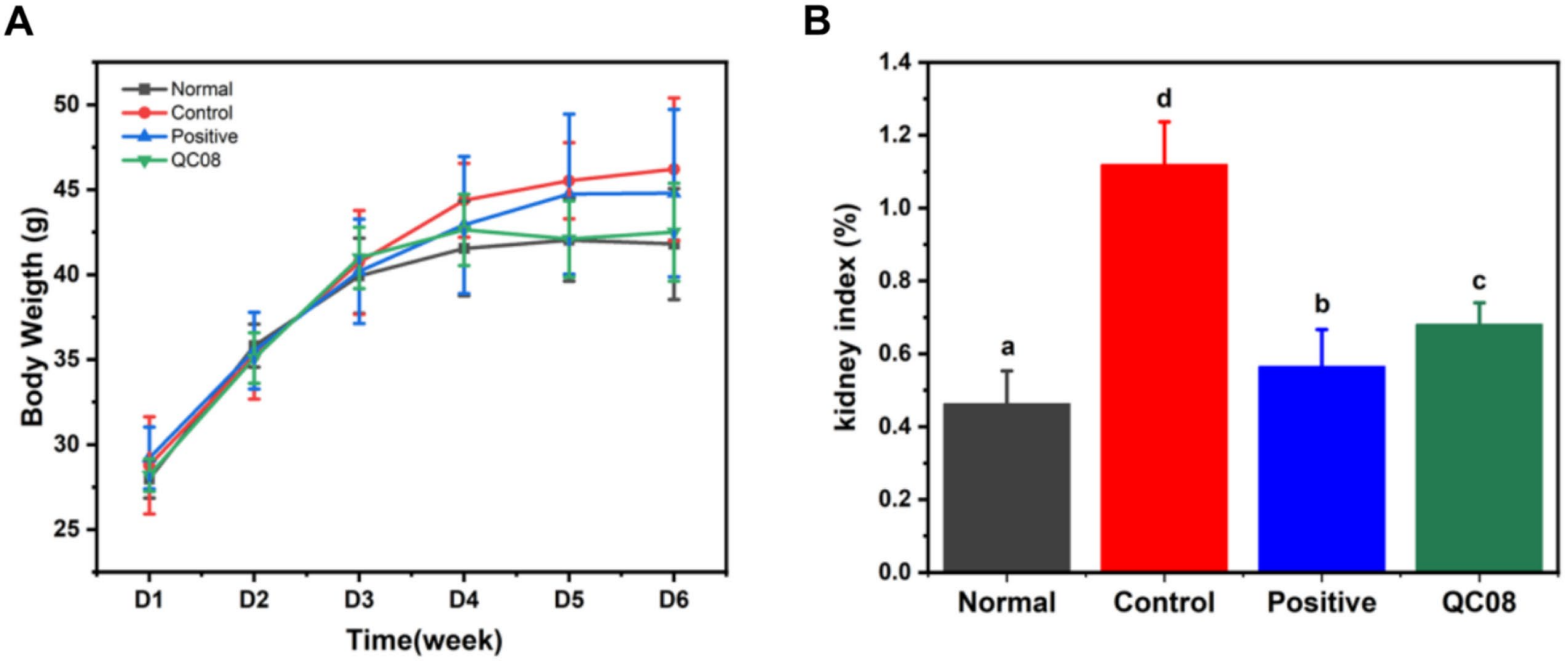
Effects of *Bifidobacterium animalis* QC08 on body weight changes **(A)** and kidney index **(B)**.

During the experiment, the body weight of mice in each group gradually increased. At the end of the experiment, there were no significant differences in body weight among the groups, indicating that the different treatments had no effect on the body weight of the mice, and probiotics exhibited no toxicity (*p* < 0.05). The kidneys play a crucial role in UA metabolism. The kidney index results indicated that the high UA model caused renal damage. Both allopurinol and *Bifidobacterium animalis* QC08 exhibited a significant inhibitory effect on kidney damage (*p* < 0.05) (Figure 1).

### Effects of *Bifidobacterium animalis* QC08 on serum levels of UA, Cr, and BUN in mice

**Figure 2:**
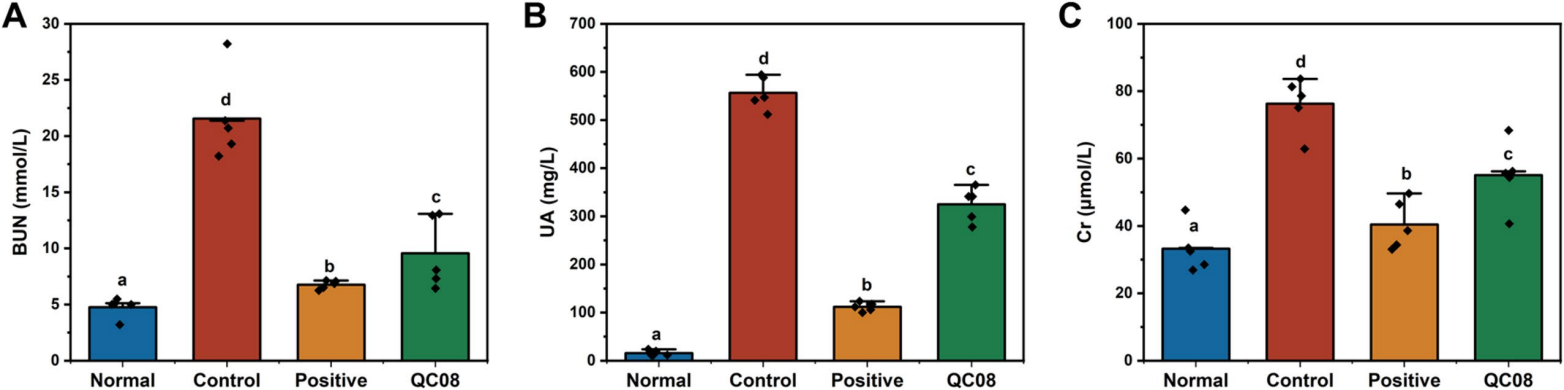
Effects of *Bifidobacterium animalis* QC08 on serum levels of BUN **(A)**, UA **(B)**, and Cr **(C)** in mice.

Yeast is abundant in nucleic acids, proteins, and other components that can elevate UA production in the body. Potassium oxonate inhibits the excretion of UA, thereby raising blood UA level. As illustrated in Figure 2, the serum levels of UA, Cr, and BUN in the control group were significantly elevated compared with the normal group (*p* < 0.05). BUN and Cr are crucial indicators of renal injury. Compared with the model group, the serum levels of UA, Cr, and BUN were significantly reduced in both the allopurinol group and the QC08 group (*p* < 0.05). This finding suggests that *Bifidobacterium animalis* QC08 could mitigate renal injury associated with the elevated blood UA level.

### Effects of *Bifidobacterium animalis* QC08 on XOD and ADA activities in the liver tissues of mice

**Figure 3:**
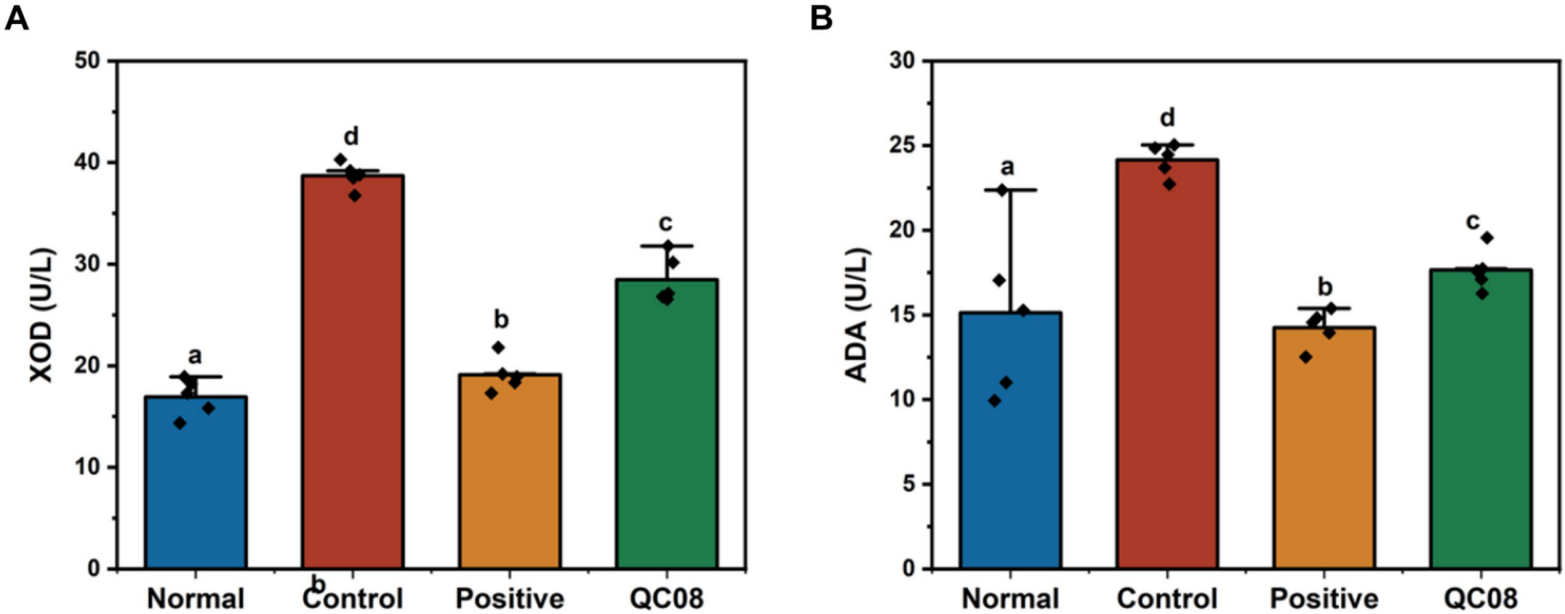
Effects of *Bifidobacterium animalis* QC08 on XOD **(A)** and ADA **(B)** activities in the liver of mice.

XOD and ADA are crucial enzymes involved in the production of UA, which are predominantly found in high concentrations in the liver and intestine. XOD catalyzes the conversion of hypoxanthine and xanthine into UA, making it a primary target for therapeutic intervention in HUA. ADA is the pivotal enzyme in the degradation of adenosine nucleotides. As illustrated in Figure 3, the hepatic activities of XOD and ADA were significantly elevated in the model group compared with the normal group (*p* < 0.05). Conversely, the activities of XOD and ADA were significantly reduced in the allopurinol and *Bifidobacterium animalis* QC08 groups compared with the model group (*p* < 0.05).

### Effects of *Bifidobacterium animalis* QC08 on serum levels of inflammatory factors (TNF-α and IL-6) in mice

**Figure 4:**
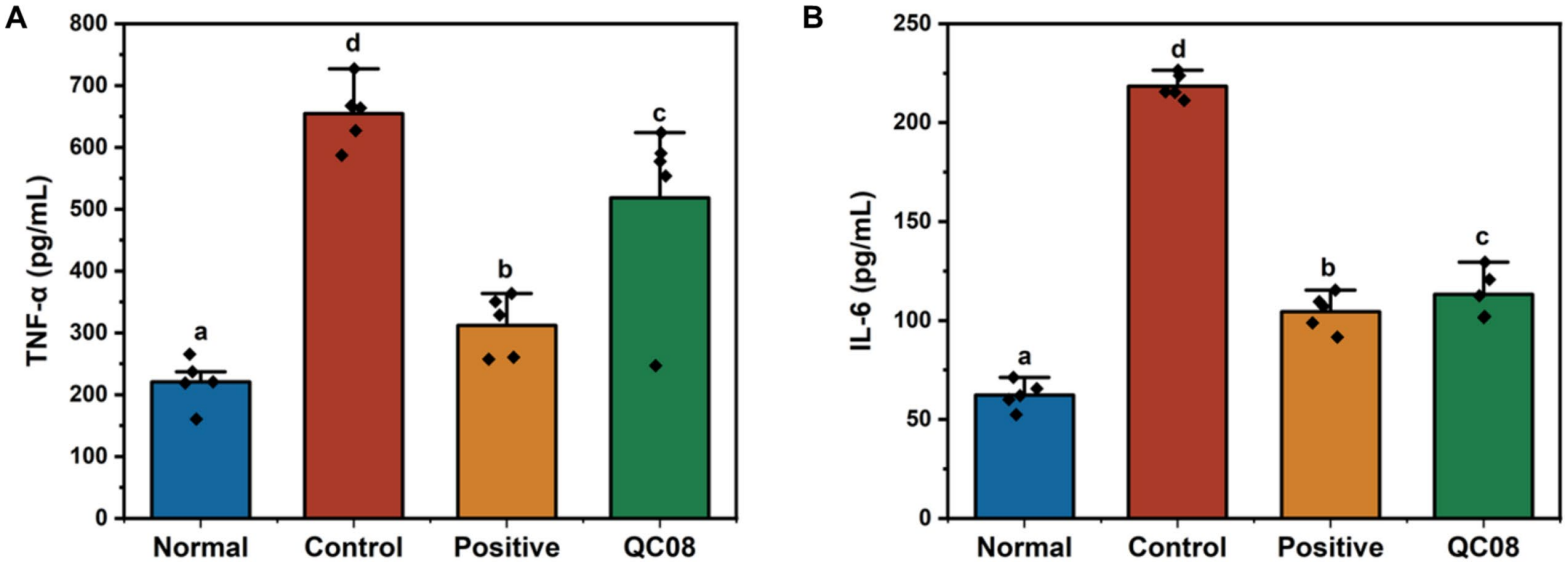
Effects of *Bifidobacterium animalis* QC08 on TNF-α and IL-6 levels in the serum of mice.

Renal injury induced by HUA is linked to systemic inflammation, which is characterized by the release of numerous inflammatory cytokines (Zhou et al., 2024). As illustrated in Figure 4, the serum levels of TNF-α and IL-6 were significantly elevated in the model group compared with the normal group (*p* < 0.05). Notably, drug intervention with allopurinol significantly reduced the levels of TNF-α and IL-6 compared with the model group (*p* < 0.05). Furthermore, the serum levels of TNF-α and IL-6 in the *Bifidobacterium animalis* QC08 group significantly decreased to 518 ± 34 and 115 ± 8 pg./mL, respectively (*p* < 0.05).

### Effects of *Bifidobacterium animalis* QC08 on histopathological changes in kidney tissue of mice

**Figure 5:**
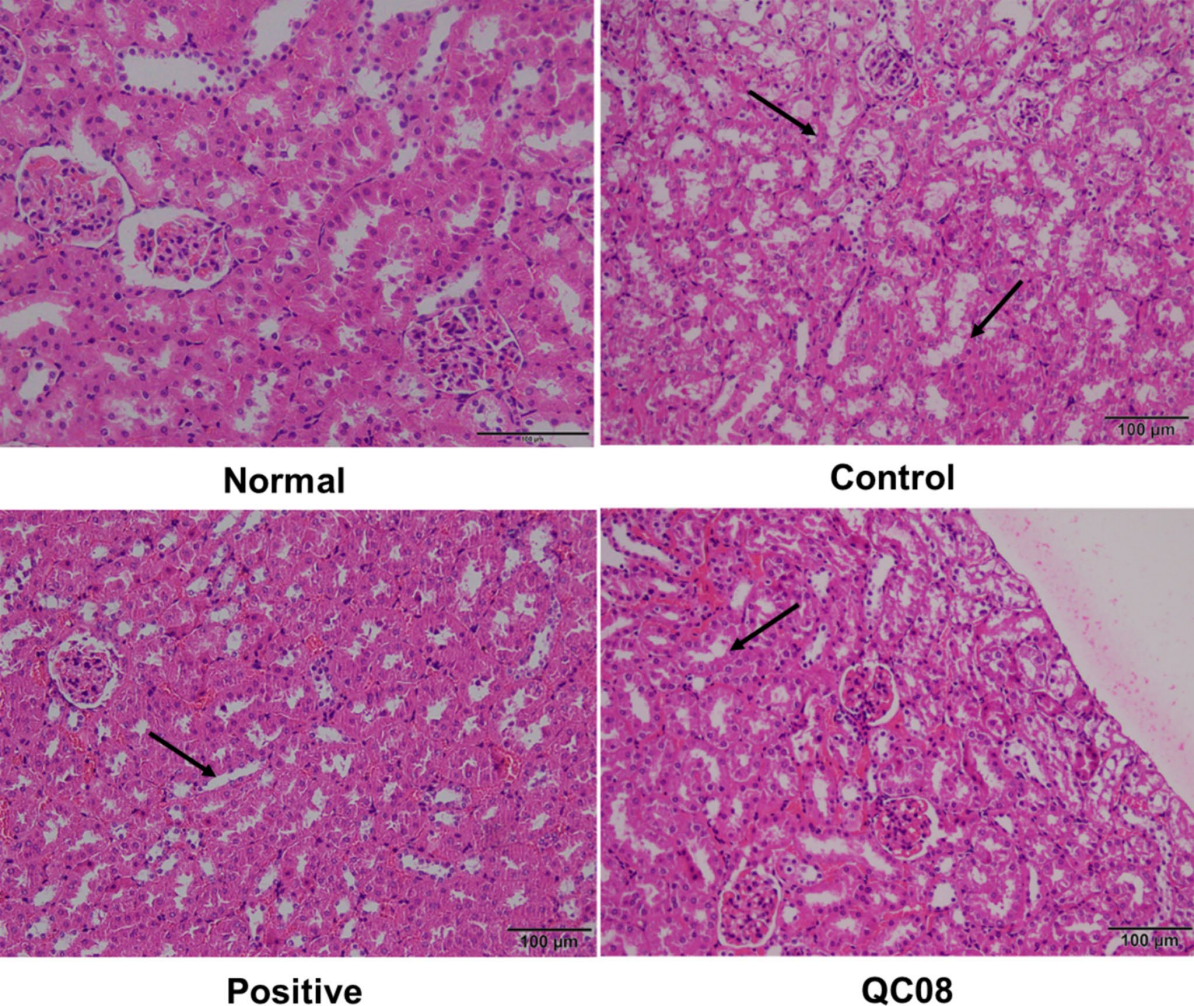
Effects of *Bifidobacterium animalis* QC08 on the histopathology of kidney tissue in hyperuricemic mice (magnification: 200×).

Figure 5 illustrates the intact kidney tissue structure in the normal group, which is characterized by normal glomerular morphology, clear boundaries, and a well-defined renal tubule lumen. In the model group, mice displayed symptoms of glomerulonephritis, including cellular infiltration, atrophy of renal tubular epithelial cells, and necrosis of renal tubular epithelium. Conversely, the kidney tissue structure in the positive control group exhibited no significant differences compared with the normal group, with the lumen recovering to levels similar to those found in the normal group. In the *Bifidobacterium animalis* QC08 group, the kidney tissue structure appeared normal, exhibiting partial recovery of the glomerular lumen and alleviation of capsule adhesion. Nonetheless, variations in the shape and size of the glomeruli persisted, suggesting that *Bifidobacterium animalis* QC08 has a protective effect on the kidney.

### Effects of *Bifidobacterium animalis* QC08 on the expression levels of OAT1, UAT, and ABCG2 genes in murine kidneys

**Figure 6:**
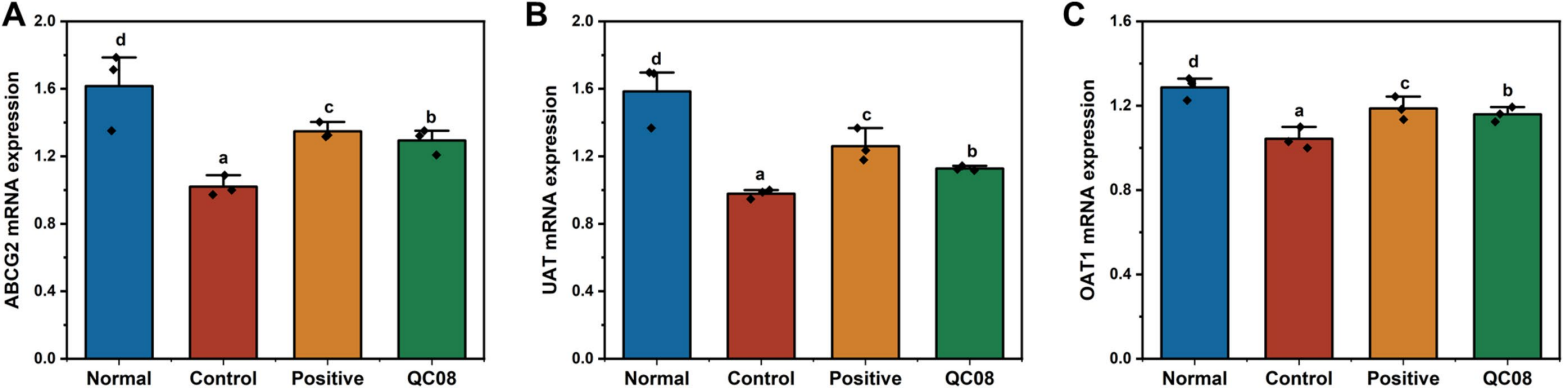
Expression levels of UAT **(A)**, ABCG2 **(B)**, and OAT1 **(C)** genes in the kidney of hyperuricemic mice.

OAT1 (organic anion transporter 1), UAT (urate transporter), and ABCG2 (ATP-binding cassette transporter G2) are critical for UA secretion, facilitating the transport of UA in the body. Figure 6 illustrates that the genes regulating the OAT1, UAT, and ABCG2 proteins were significantly downregulated in the model group compared with the normal group (*p* < 0.05). This finding indicates that the intervention involving potassium oxonate and yeast extract impaired normal UA metabolism. In the *Bifidobacterium animalis* QC08 group, the mRNA levels of UAT, OAT1, and ABCG2 significantly varied (*p* < 0.05). This suggests that *Bifidobacterium animalis* QC08 could reduce UA level in the body by upregulating the transcription of UAT, OAT1, and ABCG2 mRNA.

## Discussion

**Figure 7:**
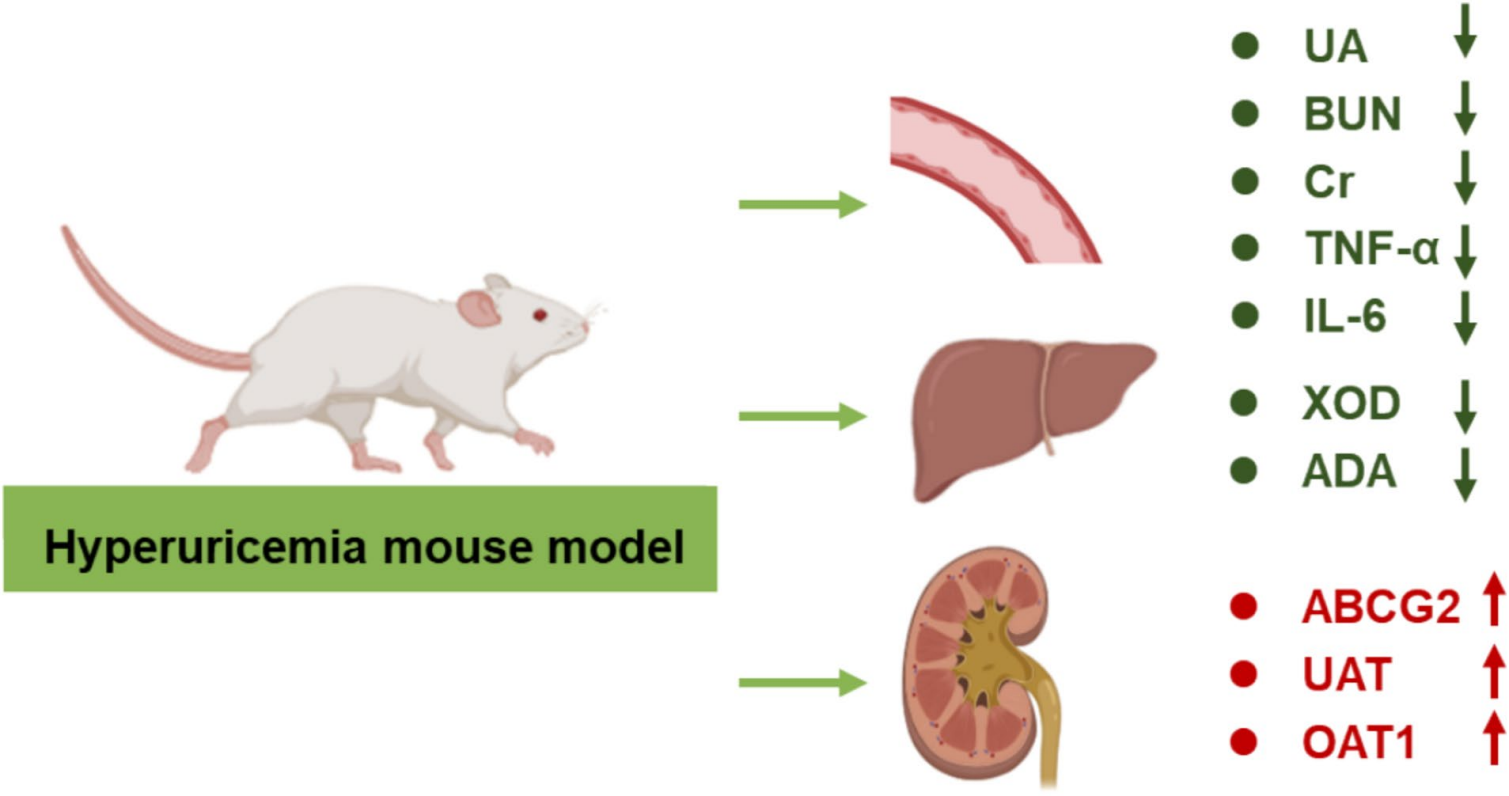
Possible mechanism of *Bifidobacterium animalis* QC08 in alleviating hyperuricemia in mice.

HUA is a metabolic disorder characterized by elevated levels of UA in the blood, leading to arthritis, hypertension, hyperlipidemia, insulin resistance, non-alcoholic fatty liver disease, and cardiovascular diseases. The medications used to treat HUA could produce serious adverse reactions. Probiotic-based therapies have gained extensive attention due to their negligible side effects. This study investigates the effects of *Bifidobacterium animalis* QC08 on the management of HUA. Possible mechanism of *Bifidobacterium animalis* QC08 was displayed in Figure 7. The excretion rate of UA decreases, leading to a higher risk of HUA (Kielstein et al., 2020). After the successful establishment of the control, the serum UA levels in *Bifidobacterium animalis* QC08-treated mice significantly decreased compared with those in the control group, indicating that *Bifidobacterium animalis* QC08 could effectively lower UA level *in vivo*. Additionally, the levels of Cr and BUN serve as critical indicators for evaluating renal filtration function. Mice in the control group exhibited significantly elevated levels of Cr and BUN, indicating that the combination of yeast extract and potassium oxonate induced renal dysfunction. Following the administration of *Bifidobacterium animalis* QC08, the serum Cr and BUN levels significantly decreased. The results suggested that *Bifidobacterium animalis* QC08 could effectively restore the levels of UA, Cr, and BUN in blood to normal levels.

The UA level is determined by the balance between hepatic synthesis and renal excretion. The UA production involves various enzymes, in which XOD is the rate-limiting enzyme in purine metabolism and plays a notable role in regulating UA level. Xanthine and hypoxanthine are oxidized by XOD to produce UA, leading to the manifestation of HUA symptoms. The XOD activity serves as an indicator of UA production. Compared with the normal group, mice in the control group demonstrated significantly elevated hepatic XOD activity. Conversely, mice in the *Bifidobacterium animalis* QC08 group exhibited a significant reduction in hepatic XOD activity compared with the control group. This suggested that *Bifidobacterium animalis* QC08 could mitigate HUA by reducing purine metabolism and UA production through the inhibition of XOD activity.

The primary mechanism for the clearance of UA is renal excretion. UA transporters (UATs) play a pivotal role in the secretion of UA in the renal proximal tubules. Compared with the normal group, the mRNA and protein expression levels of UAT were significantly reduced in the control group. In contrast, both allopurinol and *Bifidobacterium animalis* QC08 significantly upregulated the relative mRNA expression level of UAT compared with the control group. OAT1 is essential for the transport of various substances, including urate, pharmaceuticals, and other endogenous materials. Compared with the normal group, mRNA expression levels of OAT1 were significantly reduced in control group. However, treatment with allopurinol and *Bifidobacterium animalis* QC08 substantially upregulated OAT1 mRNA expression, achieving levels 1.30 and 1.16 times higher than those found in the control group, respectively. Therefore, it can be inferred that *Bifidobacterium animalis* QC08 alleviates HUA by promoting UA excretion. Studies have identified various urate transporters involved in this process. Zhu et al. found that saponins could decrease serum UA level and increase UA excretion by downregulating the expression levels of organic anion transporters (OAT1 and OAT3). Furthermore, green tea polyphenols significantly upregulated the expression levels of OAT1 and OAT3, which in turn inhibited UA reabsorption and enhanced UA secretion. ABCG2 facilitates the renal and extrarenal (intestinal) excretion of UA, and it is expressed in the apical membranes of various tissues, including the kidneys, liver, and intestines. ABCG2 functions as a primary transporter in the intestine, significantly contributing to the excretion of UA and playing a crucial role in the pathogenesis of HUA. Impaired ABCG2 activity decreases intestinal excretion of UA, resulting in the elevated serum UA level (Ichida, 2014; Ohashi et al., 2023; Yin et al., 2022). In the present study, *Bifidobacterium animalis* QC08 significantly upregulated the relative mRNA expression levels of UAT, OAT1, and ABCG2, thereby promoting UA excretion. QC08 effectively reduced UA level in the body and alleviated renal dysfunction associated with HUA through this mechanism. These findings are corroborated by the variations in serum Cr and BUN levels, alongside pathological evidence of decreased renal UA deposition.

UA can provoke inflammation in the body through both crystal-dependent and crystal-independent pathways. It stimulates activation of the inflammasome and the production of inflammatory mediators, such as IL-6 and TNF-α, which may in turn promote T cells to produce additional pro-inflammatory factors. In the present study, serum levels of IL-6 and TNF-α revealed a significant elevation in the model group compared with the normal group. Conversely, the *Bifidobacterium animalis* QC08 treatment group exhibited a significant reduction in IL-6 and TNF-α levels compared with the model group. This finding suggests that *Bifidobacterium animalis* QC08 possesses notable anti-inflammatory properties.

In summary, this study demonstrated that *Bifidobacterium animalis* QC08 could effectively reduce UA level in mice with HUA while exhibiting minimal toxicity and side effects on the kidneys. This effect may be attributed to *Bifidobacterium animalis* QC08’s capacity to inhibit hepatic XOD, thereby reducing UA synthesis, and to promote UA excretion by regulating the expression levels of urate transporters (OAT1, UAT, and ABCG2). Furthermore, *Bifidobacterium animalis* QC08 demonstrated the ability to reduce serum levels of Cr, BUN, IL-1β, and TNF-α. It could also mitigate glomerular inflammatory infiltration and damage to the glomerular epithelium, thereby enhancing renal function. These effects are likely associated with its anti-inflammatory properties. The probiotic species *Bifidobacterium animalis* QC08 exhibited potential therapeutic value in the treatment of UA-related diseases and the prevention of gout.

This study serves as a preliminary validation of the efficacy of *Bifidobacterium animalis* QC08 in the treatment of mice with HUA. It is worth noting that approximately 70% UA is eliminated via the kidneys, while the remaining 30% is excreted through the intestines. Gut microbiota could play a role in the metabolism of purines and UA. Meanwhile, the gut microbiota of gout patients exhibited significant structural and functional differences compared with those of healthy individuals. Consequently, we hypothesize that the role of *Bifidobacterium animalis* QC08 in reducing uric acid levels may be associated with underlying mechanisms of intestinal metabolism, including alterations in gut microbiota composition. Nonetheless, further research is required to validate this hypothesis.

## Data Availability

The original contributions presented in the study are included in the article/supplementary material, further inquiries can be directed to the corresponding authors.
